# Phylogenetic and metabolic diversity of Tunisian forest wood-degrading fungi: a wealth of novelties and opportunities for biotechnology

**DOI:** 10.1007/s13205-015-0356-8

**Published:** 2016-02-04

**Authors:** Dalel Daâssi, Héla Zouari-Mechichi, Lassaad Belbahri, Jorge Barriuso, María Jesús Martínez, Moncef Nasri, Tahar Mechichi

**Affiliations:** 1 Laboratory of Enzyme Engineering and Microbiology, Ecole Nationale d’Ingénieurs de Sfax, University of Sfax, Route de Soukra Km 4,5, BP 1173, 3038 Sfax, Tunisia; 2 Department of Biology, Faculty of Sciences and Arts, Khulais, University of Jeddah, Jeddah, Saudi Arabia; 3 Laboratory of Soil Biology, University of Neuchatel, Rue Emile Argand 11, 2009 Neuchâtel, Switzerland; 4 NextBiotech, Agareb, Tunisia; 5 Centro de Investigaciones Biológicas (CIB-CSIC), Ramiro de Maeztu 9, 28040 Madrid, Spain

**Keywords:** Isolation, Wood degrading fungi, Enzyme activities, Laccase, Biotechnological application

## Abstract

In this study, 51 fungal strains were isolated from decaying wood samples collected from forests located in the Northwest of Tunisia in the vicinity of Bousalem, Ain Draham and Kef. Phylogenetic analysis based on the sequences of the internal transcribed spacers of the ribosomal DNA showed a high diversity among the 51 fungal isolates collection. Representatives of 25 genera and 29 species were identified, most of which were members of one of the following phyla (Ascomycota, Basidiomycota and Zygomycota). In addition to the phylogenetic diversity, a high diversity of secreted enzyme profiles was also detected among the fungal isolates. All fungal strains produced at least one of the following enzymes: laccase, cellulase, protease and/or lipase.

**Electronic supplementary material:**

The online version of this article (doi:10.1007/s13205-015-0356-8) contains supplementary material, which is available to authorized users.

## Introduction

Wood is the most abundant biopolymer in nature and is made up mainly of cellulose, hemicellulose, and lignin. The lignin polymer is highly recalcitrant toward chemical and biological degradation due to its molecular architecture, where different non-phenolic phenylpropanoid units forming a complex three-dimensional network, are interconnected by a variety of ether and carbon–carbon bonds. Its biodegradation is a key step for carbon recycling in terrestrial ecosystems.

A number of. microorganisms including bacteria and filamentous fungi are able to degrade lignocellulosic components to various extents (Eriksson et al. 1990; Ruiz-Dueñas and Martínez 2009). Fungi participating in plant cell wall deconstruction include white rot, brown rot and leaf litter fungi (Cho et al. 2009).

The white rot fungi are the most studied because they are the unique organisms able to mineralize cell wall components with high lignin content, producing a bleached aspect of decayed wood in nature (Taylor 1995; Sigoillot et al. 2012). This ability is related to their production of a nonspecific system, including several extracellular enzymes, low molecular weight metabolites and activated oxygen species (Schoemarker 1990; Dashtban et al. 2010; Choi et al. 2012). It is worth mentioning that this system is also involved in the degradation of aromatic recalcitrant compounds causing environmental problems (Schoemarker et al. 1991; Reddy 1995).

In this sense, ligninolytic enzymes have been involved in the degradation of xenobiotic and recalcitrant aromatic compounds, industrial dyes (Khlifi et al. 2009; Daâssi et al. 2012), polycyclic aromatic hydrocarbons (PAHs) (Lee et al. 2013), pesticides (Xiao et al. 2011), dioxins (Sato et al. 2002), chlorophenols (Li et al. 2011), explosives (Cheong et al. 2006) and kraft pulp bleaching (Moldes et al. 2010).

The enzymes involved in lignin degradation include laccases, different types of peroxidases and oxidases producing H_2_O_2_ (Shimada and Higuchi 1991, Martínez et al. 2009). The most widely studied ligninolytic enzymes are, lignin peroxidase (LiP), Mn-dependent peroxidase (MnP) and laccase (Lac) (Thurston 1994; Orth and Tien 1995) in contrast to the other high redox potential peroxidases, such as versatile peroxidase (VP) and dye decolorizing peroxidase (DyP, EC 1.11.1.19) (Martínez et al. 2009; Sugano 2009). The complete conversion of lignocellulose requires the hydrolysis of structural cell wall polysaccharides by carbohydrate-acting enzymes, such as cellulases and hemicellulases (Hatakka and Hammel 2010). In addition other complementary enzymes, such as esterases/lipases or proteases, could complement the enzymatic cocktails to completely transform wood biomass into product of biotechnological interest, such as animal feed, chemical products and/or biofuel (Leonowicz et al. 1999; Barriuso et al. 2013; Dashtban et al. 2010).

Although basidiomycetes have been the most commonly studied fungi as principal organisms involved in lignin biodegradation, Ascomycetes are also able to degrade cellulose and hemicellulose despite their limitation in lignin conversion (Martínez et al. 2005). Among these ascomycetes some pathogenic fungi, such as *Fusarium solani*, are able to degrade lignin and secrete laccases and lignin peroxidases (Obruca et al. 2012).

It is estimated that there are approximately 1.5 million fungal species in the world, of which only approximately 4.6 % are described (Hawksworth, 2001). Given the wide diversity of the ligninolytic enzymes, the lignocellulosic degrading enzyme complex to which they belong and their dependence on the species and/or on the culture conditions (Hatakka, 1994; Peláez et al. 1995), the isolation and study of new ligninolytic fungi and their enzymes still receive huge interest for their putative biotechnological applications.

In this study, within the framework of a screening program of lignin microorganism degraders, we aimed to assess the fungal diversity of wood degraders in Tunisian forests. 51 new fungal species were isolated, identified, phylogenetically grouped and analyzed for their enzymes involved in biomass transformation (laccase, lipase, cellulase and protease activities).

## Materials and methods

### Chemicals

2,2′-Azino-bis(3-ethylbenzothiazoline-6-sulfonic acid) diammonium salt (ABTS) (cas: 30931-67-0), guaiacol (cas: 90-05-1) and the dyes: reactive black 5 (RB-5), Remazol Brilliant Blue R (RBBR) (cas: 2580-78-1) and Blue Turquoise (GL) (cas: 1330-38-7) were obtained from Sigma-Aldrich (Tunis, Tunisia).

### Collection of fungi

Pieces of decaying wood were collected from different habitats of the Northwest region of Tunisia (forests of Ain Draham, Bousalem and Kef) during the winters of (2009–2010) and 2011 to isolate wood-degrading fungal strains. Samples were transported in sterilized plastic bags and brought to the laboratory without further exposure to the external environment. The samples were marked with relevant information, such as number, location, and specific characteristics.

### Isolation of fungal strains

Fungal strains were isolated using a selective agar media composed of Malt Extract (ME) (30 g/L, pH 5.5) supplemented with antibiotics (ampicillin and streptomycin at 0.01 %) to avoid bacterial growth (Kiiskinen et al. 2002). The samples were surface sterilized by drying under a laminar flow hood and squares of approximately 3 × 3 mm were placed on selective agar media. The plates were subsequently incubated at 30 °C. Later on, when the mycelium had grown on the solid media, the samples were transferred to fresh agar media in Petri-dishes. This was repeated until pure cultures could be obtained as single cultures or so-called fungal isolates. Qualitative screening method for enzyme production was carried out by inoculation of 1 cm diameter pieces of mycelium from each strain onto the specific medium for the given enzyme activity.

### Enzyme activity detection in solid media

Enzymatic activities were analyzed in plates and the fungal isolates classified as positive or negative, correlated with the presence or the absence of the halo surrounding mycelium growth. To determine the activity of each isolated strain, inoculations were made in triplicate, taking a fraction (approx. 0.5 cm^2^) of the mycelium under aseptic conditions and placing it in the center of the plate containing the corresponding medium for the enzymatic activity analyzed. Then, they were incubated at 30 °C until the activity recorded.

### Detection of protease activity

The proteolytic activity of fungal isolates was tested on a milk nutrient agar medium. The medium contained 3 and 5 g/L yeast extract and peptone, respectively. The pH was then adjusted to 5.5 and the medium supplemented with 15 g/L agar.

After sterilization, 250 mL of sterile milk were added to the medium. After mixing the medium was poured into Petri dishes. The activity was determined by measuring the halo formed by the degraded milk proteins (Mayerhofer et al. 1973).

### Detection of CMCase activity

Cellulolytic activity was detected on agar plates supplemented with 1 g/L of carboxymethylcellulose (CMC). The screening medium contained (g/L): yeast extract (1.0), K_2_HPO_4_ (2.0), KH_2_PO_4_ (1.0), MgSO_4_ (1.0) MnSO_4_ (0.4), FeSO_4_ (0.01) and (NH_4_)_2_SO_4_ (3.0). The pH of the medium was then adjusted to 5.5. The plates were incubated at 30 °C and after growth, they were washed with Congo red and distained with 1 M NaCl. In positive samples a hydrolyzed zone appears transparent while the non hydrolyzed regions appeared bright red. The presence of a clear zone around the isolate demonstrated that the tested isolate produced cellulolytic enzymes (Sakthivel et al. 2010).

### Detection of lipolytic activity

Rhodamine B and olive oil agar plates (ME (30 g/L), 1 % olive oil, 0.001 % rhodamine B (Sigma, Tunis, Tunisia), and 1.5 % agar (Difco, Tunis, Tunisia) were used for the detection of lipase activity. After 7 days of incubation at 30 °C, the culture plates were revealed under 365 nm light and the activity was determined by visual evaluation of fluorescence intensity (Kouker and Jaeger 1987).

### Detection of amylase activity

The screening procedure for amylase activity detection was based on a plate culture method which uses soluble starch (1 %) as carbon source. The screening plate medium contained (g/L): yeast extract (1.0), K_2_HPO_4_ (2.0), KH_2_PO_4_ (1.0), MgSO_4_ (1.0) MnSO_4_ (0.4), FeSO_4_ (0.01) and (NH_4_)_2_SO_4_ (3.0). The pH of the medium was adjusted to 5.5 by using 12 N HCl. The medium was sterilized by autoclaving at 121 °C for 20 min. Fungi were placed on the agar medium and incubated at 30 °C for 7 days. Starch degrading fungi were identified based of the formation of clear zones after exposure with Lugol’s iodine solution (1 % iodine; 2 % potassium iodide w/v). Diameters of clear zones and fungal colonies were evaluated by a millimeter ruler (Singh and Modi 2013).

### Detection of laccase and ligninase activities

#### Primary screening: culture on solid media

To assess laccase activity, ME medium (30 g/L, 18 g/L agar) containing 4 mM guaiacol or 2 mM ABTS (2,2′-azino-di-3-ethylbenzotiazol-6-sulfonate acid) supplemented with 0.15 mM CuSO_4_ was used. Incubation of plates was performed at 30 °C. Positive laccase activity was identified by the formation of reddish brown halo in guaiacol and greenish-colored halo in ABTS supplemented plates. Observations were carried out by measuring the diameter of ring-shaped mycelia (Gnanasalomi and Gnanadoss 2013). The diameter of the halo indicated the level of laccase activity produced.

Ligninase activity was assayed based on the decolorization of different families of textile dyes such as remazol brilliant blue (RBBR), reactive black 5 (RB5) and turquoise blue (GL) (phthalocyanine). ME medium (30, 18 g/L agar) containing 150 mg/L of dye was used. Decolorization in a solid medium was assessed by visual disappearance of color from the plate (Selvam et al. 2012; Zouari-Mechichi et al. 2006).

#### Secondary screening: cultures on liquid media

The quantitative determination of laccase activity, ABTS and guaiacol oxidizing strains were grown in 500 mL Erlenmeyer flasks containing 100 mL M7 medium. Each flask was inoculated with three cylindrical plugs (4 mm in diameter) of active mycelia from previously cultured in ME agar and incubated at 30 °C, 150 rpm for 20 days. Sampling was done at regular intervals and centrifuged at 8000 rpm, +4 °C for 10 min. The supernatants were used to measure laccase activities. All the experiment was carried out in duplicates.

#### Laccase activity assay

Spectrophotometric assays of laccase activity were carried out with 1 mM guaiacol or 10 mM 2,2′-azino-bis-(3-ethylbenzthiazolinesulphonate) (ABTS) as substrates, in 100 mM sodium acetate buffer (pH 5.0). When guaiacol was used, the absorbance was monitored at 465 nm (*ε* = 12,000 M^−1^ cm^−1^); when ABTS was the substrate, the absorbance was monitored at 436 nm (*ε* = 29,300 M^−1^ cm^−1^) (Silva et al. 2005).

### Identification of fungal isolates by sequencing the ITS region

Each isolate was cultivated in a 100 mL flask containing 25 mL of liquid ME medium for 5 days. Then mycelium was collected, washed with sterile Milli-Q water and lyophilized.

The molecular identification was carried out with the protocol presented by Daâssi et al. (2013). The primers used for the amplification were ITS1 (5′-TCCGTAGGTGAACCTGCGG-3′) and ITS4 (5′-TCCTCCGCTTATTGATATGC-3′) (White et al., 1990). Sequencing was performed using an automated ABI Prism 3730 DNA sequencer (Applied Biosystems, Tunis, Tunisia).

### DNA sequence and phylogenetic analysis

The resulting sequences were subjected to BlastN analysis (www.ncbi.nlm.nih.gov/BlastN). The organisms were identified relying on the sequences available in the database presenting the highest homology. The sequences from the different isolated strains and those from the GenBank were aligned using CLUSTAL W (Thompson et al. 1994). Distances were calculated and phylogenetic trees were constructed using the neighbour-joining method (Saitou and Nei, 1987). Bootstrap analysis was done based on 1000 replicates. The MEGA3 package was used for all analyses. Sequences have been deposited in GenBank. Genbank accession numbers will be provided later.

## Results and discussion

### Isolation and identification of fungi isolated from Tunisian forests

The isolation experiments resulted in fifty-one fungal isolates. The internal transcribed spacer (ITS) regions of fungal rDNA have been successfully used for species identification (Table 1). The fungal strains isolated during this work were found to be highly diverse based on the ITS genetic marker (Table 2). The capacity of the isolates to produce laccases, lipases, proteases, amylases, and cellulolytic enzymes was investigated on selective solid media (Table 3).

**Table 1:** Blast report showing the accession no and total score query coverage max identity

No.	Strain	Molecular identification	Accession No	Max identity (%)
1	BS54	*Coriolopsis gallica*	gb|AY684172.1|	99
2	BS2	*Phanerochaete chrysosporium*	gb|JQ796876.1|	100
3	BS22	*Coriolopsis trogii*	gb|HM989941.1|	99
4	TM11	*Bjerkandera adusta*	gb|FJ608590.1|	99
5	BS17	*Byssochlamys spectabilis*	gb|FJ895878.1|	100
6	BS34	*Ganoderma carnosum*	gb|JQ520163.1|	99
7	D11	*Trichoderma harzianum*	gb|HQ596939.1|	100
8	D6	*Ilyonectria destructans*	emb|AJ875317.1|	99
9	BS14	*Coniochaeta * sp.	gb|JN225913.1|	99
10	BS19	*Neurospora * sp.	gb|JF749204.1|	99
11	11B	*Bjerkandera adusta*	gb|JF439464.1|	99
12	A3	*Trametes versicolor*	gb|KC492579.1|	94
13	BS56	*Trichoderma citrinoviride*	gb|KC009820.1|	100
14	AD40	*Coriolopsis trogii*	gb|JN164998.1|	98
15	BS25	*Porostereum spadiceum*	gb|JX46661.1|	80
16	CLBE1	*Phoma macrostoma*	gb|JF723492.1|	82
17	CLBE2	*Spencermartinsia viticola*	gb|AY905555.1|	100
18	CLBE3	*Fusarium solani*	gb|EU750688.1|	99
19	CLBE4	*Fusarium oxysporum*	gb|AF322074.1|	100
20	CLBE5	*Fusarium equiseti*	gb|EU326202.1|	99
21	CLBE6	*Alternaria tenuissima*	gb|EU326185.1|	100
22	CLBE9	*Peyronellaea glomerata*	gb|AY183371.1|	100
23	CLBE10	*Trichoderma gamsii*	gb|JQ040342.1|	100
24	CLBE11	*Fusarium equiseti*	gb|AY147362.1|	100
25	CLBE12	*Alternaria alternata*	gb|GQ121322.2|	99
26	CLBE13	*Trichoderma harzianum*	gb|EU280078.1|	99
27	CLBE14	*Mucor racemosus*	gb|HM641690.1|	99
28	CLBE15	*Gloeophyllum trabeum*	emb|AJ420949.1|	99
29	CLBE16	*Gloeophyllum trabeum*	emb|AJ420949.1|	99
30	CLBE17	*Alternaria *sp.	gb|EF432274.1|	100
31	CLBE18	*Trichoderma harzianum*	gb|EU280078.1|	100
32	CLBE19	*Fusarium equiseti*	gb|EU326202.1|	100
33	CLBE20	*Trichoderma viride*	gb|AF455432.1|	99
34	CLBE24	*Uncultured fungus.*	emb|FN689670.1	99
35	CLBE29	*Thermomyces lanuginosus*	dbj|AB085929.1|	100
36	CLBE33	*Fusarium solani*	gb|JQ277276.1|)	99
37	CLBE35	*Thermomyces lanuginosus*	dbj|AB085929.1|	94
38	CLBE40	*Thermomyces lanuginosus*	dbj|AB085929.1|	100
39	CLBE41	*Thermomyces lanuginosus*	dbj|AB085929.1|	100
40	CLBE44	*Thermomyces lanuginosus*	dbj|AB085929.1|	100
41	CLBE47	*Thermomyces lanuginosus*	gb|JQ639282.1	99
42	CLBE49	*Coniochaeta * sp.	gb|JN225913.1|	99
43	CLBE50	*Preussia minima*	gb|DQ468027.1|	99
44	CLBE51	*Cladosporium cladosporioides*	gb|JX981454.1|	99
45	CLBE53	*Coniochaeta* sp.	gb|JN225913.1|	99
46	CLBE54	*Perenniporia medulla*-*panis*	gb|JQ673013.1|	99
47	CLBE55	*Coriolopsis trogii*	gb|HM989941.1|	100
48	CLBE56	*Phanerochaete chrysosporium*	dbj|AB361644.1|	99
49	CLBE57	*Phanerochaete chrysosporium*	gb|KP135093.1|	99
50	CLBE58	*Alternaria brassicae*	gb|JX290150.1|	99
51	CLBE59	*Trichoderma harzianum*	gb|JF923802.1|	99

**Table 2:** Number of fungal genera and species among different taxonomic groups in Tunisian forests and their relative load (%)

Phylum	Orders	Number of isolates	Number of genera	Number of species	Load %
Uncultured fungus	–	1	1	1	2
Mucoromycotina	Mucorales	1	1	1	2
Ascomycetes	Coniochaetales	3	1	1	6
	Pleosporales	7	3	8	14
	Capnodiales	1	1	1	2
	Eurotiales	7	2	2	14
	Botryosphaeriales	1	1	1	2
	Sordiariales	1	1	1	8
	Hypocreales	14	3	8	27
Basidiomycetes	Polyporales	13	7	8	25
	Gloeophyllales	2	1	2	4
Total number of isolates	–	51	25	29	100
Load %	–	100	49	57

**Table 3:** Screening of guaiacol-oxidation activity and molecular identification for different isolates

No.	Strains	Enzymatic activities					Molecular identification
		Lac^1^	CMCas^2^	Prot^3^	Amyl^4^	Lip^5^	
1	BS54	+++	++	–	++	+	*Coriolopsis gallica*
2	BS2	++	–	–	++	–	*Phanerochaete chrysosporium*
3	BS22	++	+	+	++	–	*Coriolopsis trogii*
4	TM11	+++	–	+	–	–	*Bjerkandera adusta*
5	BS17	+	+++	–	+++	–	*Byssochlamys spectabilis*
6	BS34	+++	–	+	–	–	*Ganoderma carnosum*
7	D11	–	++	–	–	–	*Trichoderma harzianum*
8	D6	+	++	+++	+	+++	*Ilyonectria destructans*
*9*	*BS14*	+	++	+++	–	–	*Coniochaeta* sp.
*10*	*BS19*	+	++	–	+++	+	*Neurospora *sp.
11	11B	+++	+	–	–	+	*Bjerkandera adusta*
12	A3	+++			–	–	*Trametes versicolor*
13	BS56	–	+++	–	–	–	*Trichoderma citrinoviride*
14	AD40	+++	–	+	++	–	*Coriolopsis trogii*
15	BS25	+++	+	–	++	–	*Porostereum spadiceum*
16	CLBE1	+	–	+	–	+	*Phoma macrostoma*
17	CLBE2	+	+	–	–	+	*Spencermartinsia viticola*
18	CLBE3	–	+	–	–	+	*Fusarium solani*
19	CLBE4	–	+	–	–	+++	*Fusarium oxysporum*
20	CLBE5	–	+	–	–	+++	*Fusarium equiseti*
21	CLBE6	–	–	+	+	+	*Alternaria tenuissima*
22	CLBE9	–	–	–	–	+	*Peyronellaea glomerata*
23	CLBE10	–	+++	–	–	+	*Trichoderma gamsii*
24	CLBE11	–	–	+	–	+++	*Fusarium equiseti*
25	CLBE12	–	+	–	–	–	*Alternaria alternata*
26	CLBE13	–	++	–	–	–	*Trichoderma harzianum*
27	CLBE14	++	–	–	–	–	*Mucor racemosus*
28	CLBE15	++	++	+++	–	–	*Gloeophyllum trabeum*
29	CLBE16	++	++	+++	+	–	*Gloeophyllum trabeum*
30	CLBE17	–	+	++	–	–	*Alternaria *sp.
31	CLBE18	–	++	–	+	–	*Trichoderma harzianum*
32	CLBE19	–	–	–	–	+++	*Fusarium equiseti*
33	CLBE20	–	+++	–	–	+	*Trichoderma viride*
34	CLBE24	–	–	–	–	–	*Unculture fungus* sp.
35	CLBE29	+	–	+	–	+	*Thermomyces lanuginosus*
36	CLBE33	–	–	–	+	+	*Fusarium solani*
37	CLBE35	++	–	–	–	+	*Thermomyces lanuginosus*
38	CLBE40	++	–	–	–	+	*Thermomyces lanuginosus*
39	CLBE41	++	–	–	–	–	*Thermomyces lanuginosus*
40	CLBE44	++	–	–	–	+	*Thermomyces lanuginosus*
41	CLBE47	–	–	+	+	+	*Thermomyces lanuginosus*
42	CLBE49	++	–	+	–	+	*Coniochaeta *sp.
43	CLBE50	–	+	–	–	+	*Preussia minima*
44	CLBE51	++	–	+	+	+	*Cladosporium cladosporioides*
45	CLBE53	++	–	+	–	–	*Coniochaeta *sp.
46	CLBE54	+++	+	++	+	–	*Perenniporia medulla*-*panis*
47	CLBE55	++	–	+	–	–	*Coriolopsis trogii*
48	CLBE56	+	–	–	+	++	*Phanerochaete chrysosporium*
49	CLBE57	++	–	++	+	+	*Phanerochaete chrysosporium*
50	CLBE58	++	–	+	+	+	*Alternaria brassicae*
51	CLBE59	–	++	–	–	–	*Trichoderma harzianum*

Lac, laccase activity; CMCase, cellulase activity; Prot, protease activity; Amyl, amylase activity; Lip, lipase activity

(+) Diameter of the halo surrounding mycelium growth 0–15 mm, (++) halo diameter 15–25 mm, (+++) halo diameter up to 25 mm

The levels of similarity (Table 1) allowed us to assign all fungal isolates to the genus or even the species level according to Rossello Mora and Amman (2001) except few isolates such as isolate CLBE 24 that matched an undescribed uncultured fungus. The 51 fungal isolates belong to 22 genera and 34 species (Table 2). Ascomycete fungi of the genera: *Phoma* (single strain), *Ilyonectria* (1 strain), *Coniochaeta* (3 strains), *Neurospora* (1 strain), *Trichoderma* (7 strains), *Spencermartinsia* (1 strain), *Fusarium* (6 strains), *Alternaria* (4 strains), *Thermomyces* (6 strains), *Preussia* (1 strain), *Cladosporium* (1 strain), *Paecilomyces* (1 strain), *and Pyronellaea* (1 strain), basidiomycetes of the genera: *Trametes* (1 strain), *Coriolopsis* (4 strains), *Phanerochaete* (3 strains), *Ganoderma* (1 strain), *Bjerkandera* (2 strains), *Gloeophyllum* (2 strains), *Prosterium* (1 strain) and *Perenniporia* (1 strain) and Mucoromycetes of the genus *Mucor* (1 strain), were identified and studied in detail.

### Secreted enzymatic activities

All 51 isolates were screened by plate test methods for laccases, proteases, amylases, cellulases, and lipases production. Data presented in Table 3 shows that among the tested isolates, 32 strains were found to produce laccases, 25 strains were found to produce cellulases, 18 strains were found to produce amylases, 21 strains were found to produce proteases and 27 strains were found to produce lipases. Therefore, the results of enzymes screening (Table 3) show a high diversity of enzymatic activities in the fungi isolated from Tunisian forests. Several studies reported that ligninolytic fungi use a combination of several activities (hydrolases, oxidoreductases and esterases) for wood degradation indicating the complexity of the ligninocellulosic materials (Yoon et al. 2007; Ljungdahl 2008; Dashtban et al. 2010).

### Protease activity producing fungi

Table 3, clearly shows that protease activity is revealed principally in basidiomycete taxa, including fungi from *Trametes*, *Ganoderma* and *Phanerochaete* genera although ascomycete fungi, from *Ilyonectria* genus, were able to produce this activity. While, the presence of extracellular proteases in basidiomycete fungi, such as *Coriolopsis trogii* (Caporale et al. 1996) and *Trametes versicolor* (Staszczak and Nowak, 1984), has been well known for decades, their occurrence in *Phanerochaete chrysosporium* is a current topic of interest (Dass et al. 1995; Xiong et al. 2008). These authors investigated the effect of nitrogen excess/limitation culture conditions on the expression of proteases and peroxidases in *P. chrysosporium*. They reported that the wood-degrading fungus *P. chrysosporium* produces proteolytic activity associated to excess nitrogen conditions (non-ligninolytic conditions). However, when subjected to nitrogen limitation, *Phanerochaete* produces principally peroxidases to depolymerize lignin in wood. These studies provide clues to optimize extracellular enzymes production by fungi in synthetic media for biotechnological applications.

### Lipase activity producing fungi

Lipolytic activity has been studied in the isolates collection. The results (Table 3) reveal a relevant capacity of the tested fungi to secrete lipase, making their further study very interesting. Among the tested strains, 27 lipase-producing fungi were detected within ascomycete group, such as *Thermomyces*, *Fusarium* and *Neonectria*. In *Fusarium* and *Thermomyces* the capacity to synthesize this type of enzymes is well characterized (Jallouli et al. 2012; Kovalenko et al. 2013). Our Results also agree with those of Cruz-Ramírez et al. (2012), where 49 lipase producing fungi have been recovered from a screening of fungal isolates from decaying wood samples collected in Mexico.

### Cellulase producing fungi

Table 3 shows that among the 51 decay-wood fungi isolates, 25 strains present degradation halos reflecting a good activity on carboxymethylcellulose (CMCase) activity, indicating that this fungus is able to secrete a high β-1,4-endoglucanase activity. It is well known that fungal lignin degradation involves lignocellulolytic enzymes such as CMCases to degrade lignocellulosic components in wood. CMCases are important enzymes necessary for the degradation of cellulose and have been reported from well-known strains of *Trichoderma* and *Paecilomyces* (Colussi et al. 2011; Hussain et al. 2012). The ability of these fungal strains to degrade lignocellulosic biomass, as well as their utility in treatments of pulp and paper mill effluent, have been mentioned in many reports (Hussain et al. 2012). In addition to CMCase other cellulolytic enzymes such as cellobiohydrolases were shown to be also secreted simultaneously in *Trichoderma harzianum IOC 3844* (de Castro et al. 2010; Colussi et al. 2011) and *Paecilomyces variotii 103*-*7* isolated from naturally degraded wood (Hussain et al. 2012).

### Amylase producing fungi

Amylase activity was detected in some members of the fungal collection and was present in 35 % of the total isolates. *P. variotii* and *Neurospora* sp. were good producers of amylases (Table 3). In the same trend, Michelin et al. (2010) reported the purification of a thermostable α-amylase produced by the fungus *P. variotti*.

### Laccase producing fungi

Wood decay fungi are a very good source of lignin enzymes typically phenol oxidases (laccases). Data presented in Table 3 show that of the 51 tested strains, 31 exhibited significant guaiacol-oxidation activity expressed within the first week of incubation. Most of basidiomycetes were able to produce laccase and among them representatives of the genera *Trametes*, *Phanerochaete*, *Ganoderma*, *Bjerkandera,* and *Porostereum* were ranked as the best producers (Table 3). The basidiomycetous white-rot fungi are the most efficient lignin-degrading organisms in nature (Fackler et al. 2006).

### Primary screening: culture on solid media

Fungi showing guaiacol-oxidation activities in Table 3 were cultivated on solid media containing 4 mM guaiacol or 2 mM ABTS in the presence of 150 mM CuSO_4_ that enabled the detection of laccases as specific color reactions (Machado et al. 2005). Guaiacol and ABTS were considered as the best substrates for laccase activity (Thurston 1994).

**Fig. 1:**
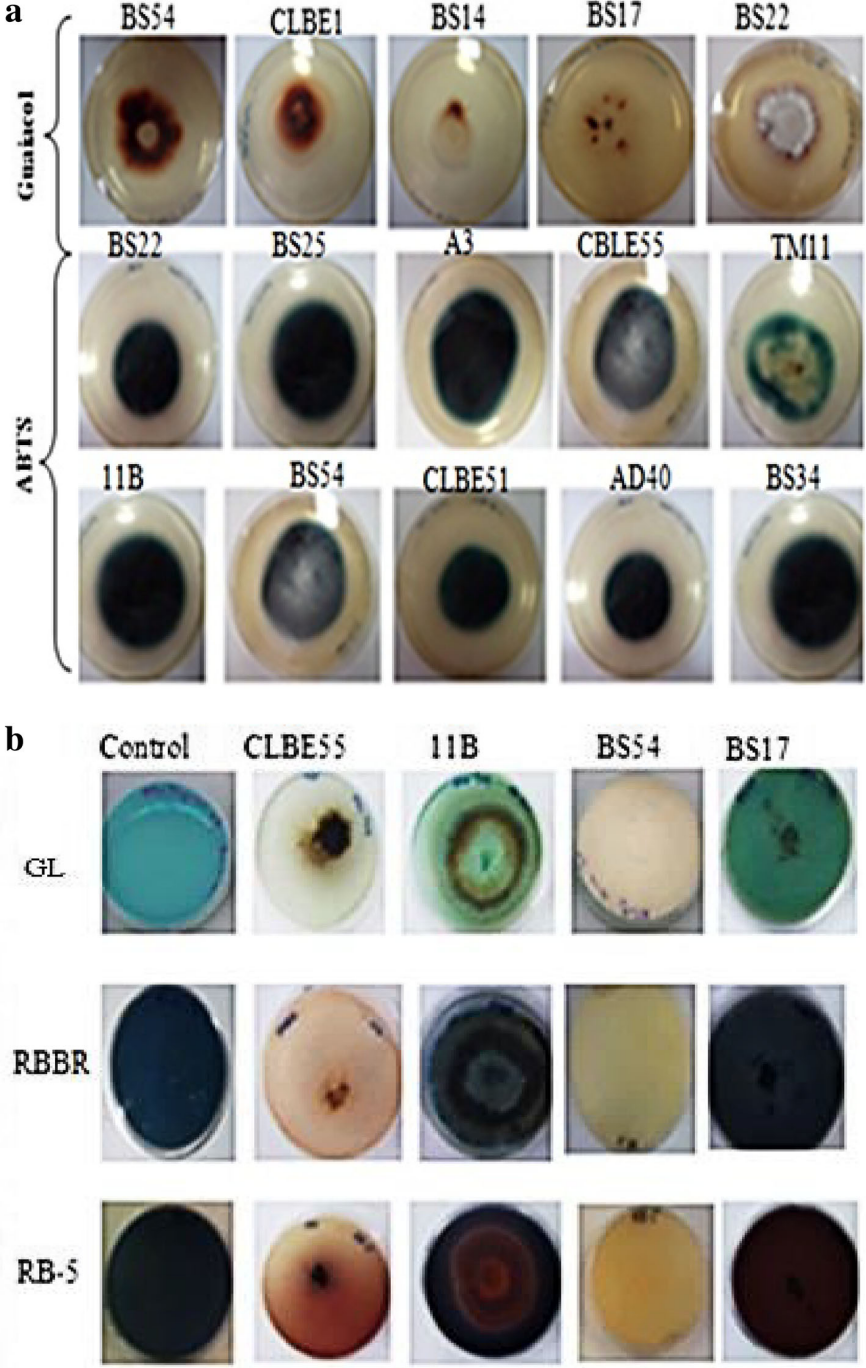
Photo of ME agar plate showing positive guaiacol, ABTS (**a**) and dyes (**b**) (GL, RB-5 and RBBR) oxidation by fungal isolates obtained from decayed wood samples after 7 days of inoculation

The positive strains showed reddish brown halo in guaiacol supplemented agar and dark-green halo in ABTS supplemented plates (Fig. 1a). The diameter of the halo and the color intensity indicating a positive extracellular oxidoreductase secretion from mycelium was used to screen the level of laccase production of each strain (Table 4).

**Table 4:** Qualitative test for Lac+ isolates using colored indicators (4 mM of guaiacol and 2 mM of ABTS) and quantitative test in liquid M7 medium (with their 95 % confidence limits; Mean ± standard deviation (*n* = 3); 30 °C; 150 rpm, in the presence of 150 mM CuSO_4_)

No.	Strains	Qualitative test		Quantitative test	
		Reddish-brown zone formation (guaiacol)	Green halo zone formation (ABTS)	Lac activity (U/L) with guaiacol	Lac activity (U/L) with ABTS
1	BS54	+++	++	3880 ± 12.2 (9 day)	4761 ± 5.3 (9 day)
2	BS2	++	+++	ND	–
3	BS22	++	+++	1256 ± 6.4 (9 day)	1443 ± 3.5 (9 day)
4	TM11	+++	++	4545 ± 8.2 (9 day)	4914 ± 4.7 (9 day)
5	BS17	+	+	–	–
6	BS34	+++	++	1556 ± 6.1 (6 day)	1788 ± 7.2 (6 day)
7	D6	+	+	94 ± 3.1 (4 day)	123 ± 1.9 (4 day)
8	BS14	+	+	864 ± 9.2 (5 day)	1127 ± 6.8 (5 day)
9	BS19	+++	+	122 ± 3.6 (9 day)	165 ± 1.9 (9 day)
10	11B	+++	+++	8358 ± 14.7 (12 day)	11,134 ± 10.3 (12 day)
11	A3	++	+++	5233 ± 6.5 (8 day)	7064 ± 9.1 (8 day)
12	AD40	++	+++	4276 ± 11.4 (8 day)	5855 ± 6.7 (8 day)
13	BS25	+	++	1120 ± 3.1 (6 day)	1252 ± 1.9 (6 day)
14	CLBE1	++	++	1944 ± 5.7 (6 day)	2054 ± 9.3 (6 day)
15	CLBE2	++	++	2031 ± 12.6 (6 day)	2645 ± 10.5 (6 day)
16	CLBE14	++	+	723 ± 5.3 (6 day)	789 ± 2.3 (6 day)
17	CLBE15	+		ND	ND
18	CLBE16	+	+	ND	ND
19	CLBE29	+	+	22 ± 9.6 (6 day)	88 ± 1.3 (6 day)
20	CLBE35	+	+	34 ± 2.3 (4 day)	54 ± 6.4 (4 day)
21	CLBE40	+		76 ± 4.5 (5 day)	126 ± 2.7 (5 day)
22	CLBE41	++	+	50 ± 1.3 (6 day)	102 ± 6.3 (6 day)
23	CLBE44	++	+	52 ± 1.7 (5 day)	87 ± 0.7 (5 day)
24	CLBE49	+++	+	401 ± 2.6 (6 day)	654 ± 5.2 (6 day)
25	CLBE51	++	+	565 ± 2.6 (7 day)	843 ± 1.3 (7 day)
26	CLBE53	++	+	774 ± 2.6 (6 day)	1123 ± 5.5 (6 day)
27	CLBE54	+++	+	454 ± 2.6 (17 day)	732 ± 3.4 (17 day)
28	CLBE55	+++	+++	20,000 ± 9.6 (12 day)	24,233 ± 6.2 (12 day)
29	CLBE56	+	+	ND	ND
30	CLBE57	+	+	ND	ND
31	CLBE58	+	+	122 ± 1.8 (9 day)	176 ± 2.1 (9 day)

*ND* not detected laccase activity

(+) Diameter of the oxidized zone 0–15 mm, (++) zone diameter 15–25 mm, (+++) zone diameter up to 25 mm

### Secondary screening: cultures on liquid media

Laccase production by the fungal strains that were positive in the plate-test screening was further studied in liquid cultures.

Secondary screening experiments with the selected isolates showed that the activity of laccase was higher with ABTS than with guaiacol. Else, the highest levels of laccase production were produced by the basidiomycetous especially white-rot fungi which confirms the results find in the qualitative test. The white-rot basidiomycete *C. trogii* (CLBE55) was isolated from decayed acacia wood from North West of Tunisia and selected for its potential of laccase production (Zouari-Mechichi et al. 2006). These results are in agreement with those of Gnanasalomi and Gnanadoss (2013) who reported the isolation of twenty-six basidomycetes that produce laccases.

As presented in Table 4, some fungal strains show laccase activity in a solid medium but not when cultured in a liquid medium. This may be explained by the need to create the proper conditions for laccase production for each isolate. In fact, the liquid medium (M7) was supported laccase production by basidiomycetes more than others taxonomic groups (Zouari-Mechichi et al. 2006). Some groups require such element in the cultures for laccase secretion.

A similar trend toward laccase activity dependence on the cultivation medium and conditions was reported by Kiiskinen et al. (2004) and Colao et al. (2003).

On the contrary Ryu et al. (2003) explain the observed difference between the revelation of laccase activity in solid and liquid medium by the fungal strains may have produced other ligninolytic enzymes.

To demonstrate the potential for biotechnological applications of our laccase producing fungi, we used them in further experiments to select ligninolytic fungal isolates able to decolorize and detoxify textile dyes, a relevant effluent produced by Tunisian textile industries and an important pollutant in natural ecosystems in Tunisia (Zouari-Mechichi et al. 2006).

### Laccase-producing fungi with potential for textile dye decolorization

Ligninolytic systems, especially laccases, are directly involved in the degradation of various xenobiotic compounds and dyes. Their capacities to remove and decolorize dyes from textile industry effluents make them a useful tool for bioremediation purposes (Zouari-Mechichi et al. 2006).

As previously mentioned, 63 % of laccase-producing fungi were found among the fungal isolates by qualitative test on plates. The ligninolytic activity of the 31 laccase-producing fungi was further confirmed by their ability to oxidize three commercial industrial dyes (RBBR, RB-5 and GL) (Fig. 1b). It was observed that all screened isolates positive with guaiacol were able to oxidase RBBR and GL. The correlation between the polymeric dyes and guaiacol was also good, as only one strain, BS17 (*Byssochlamys spectabilis*), was positive on guaiacol without being positive on polymeric dye indicators (Table 5). Our results suggest that color reactions with RBBR, GL and guaiacol are more easily detectable and these compounds can reliably be used for laccase activity screening. Moreover, strains able to oxidize the three dyes: anthraquinonic (RBBR), diazoic(RB-5) and phthalocyanine (GL), correlated well with each other in the oxidation of ABTS or guaiacol (diameter >15 mm). Some other strains were able to decolorize RBBR and GL but not RB-5 such as *T. harzianum* D11, *Ilyonectria destructans* D6, *Coniochaeta* sp. BS14, *Phoma macrostoma* CLBE1, *Spencermartinsia viticola* CLBE2, *Gloeophyllum trabeum* CLBE16, *Thermomyces lanuginosus* CLBE29, *Perenniporia medulla*-*panis* CLBE54, *Phanaerochaete chrysosporium* CLBE56 and CLBE57 and *Alternaria brassicae* CLBE58. This correlates with the recalcitrant nature of RB-5 as diazoic dye and the need of system-laccase mediator (SLM) for RB-5 degradation as reported in many studies (Daâssi et al. 2012).

**Table 5:** Comparison of reactions of the isolated fungal strains with different indicators (4 mM guaiacol and dyes: RBBR, RB-5 and GL) on malt extract agar plates

No.	Fungal isolates	Mycelial growth and oxidation characteristics			Oxidation of dyes		
		Colour zone diameter (mm)^a^	Oxidation scale^b^	Fungal colony diameter(mm)^c^	RBBR	RB-5	GL
1	BS54	22	++	29	*	*	*
2	BS2	21	++	30	*	*	*
3	BS22	15	++	29	*	*	*
4	TM11	21	++	18	*	*	*
5	BS17	10	+	33	–	–	–
6	BS34	25	+++	29	*	*	*
8	D6	8	+	12	*	–	*
9	BS14	10	+	17	*	–	*
10	BS19	32	+++	25	*	*	*
11	11B	31	+++	30	*	*	*
12	A3	21	++	29	*	*	*
13	AD40	24	++	30	*	*	*
14	BS25	12	+	18	*	*	*
15	CLBE1	15	++	20	*	–	*
16	CLBE2	18	++	25	*	–	*
17	CLBE14	21	++	27	*	*	*
18	CLBE15	12	+	17	*	*	*
19	CLBE16	10	+	18	*	–	*
20	CLBE29	14	+	19	*	–	*
21	CLBE35	12	+	18	–	*	–
22	CLBE40	14	+	20	–	*	–
23	CLBE41	18	++	22	–	*	–
24	CLBE44	20	++	18	*	–	–
25	CLBE49	21	+++	25	*	*	*
26	CLBE51	18	++	15	*	*	*
27	CLBE53	16	++	24	–	–	–
28	CLBE54	32	+++	24	*	–	*
29	CLBE55	22	+++	14	*	*	*
30	CLBE56	10	+	17	*	–	*
31	CLBE57	9	+	18	*	–	*
32	CLBE58	10	+	22	*	–	*

^a^Diameter of the oxidized zone in mm (measured on the 7th day of cultivation)

^b^Oxidation scale measured on the 7th day of cultivation on selective- medium containing 4 mM guaiacol

^c^Diameter of the mycelial colony in mm measured on the 7th day of cultivation (the initial disc 10 mm diameter)

(+) Diameter of the oxidized zone 0–15 mm, (++) zone diameter 15–25 mm, (+++) zone diameter up to 25 mm, (–) absence of clarified zone, (*) presence of clarified zone

Decolorization of synthetic dyes has been demonstrated using white rot basidiomycetes (Diwaniyan et al. 2010; Daâssi et al. 2013) such as *P. chrysosporium* (Gomaa, 2012), *Ganoderma* sp. (Ma et al. 2014 *)*, *Trametes trogii* (Zouari-Mechichi et al. 2006), *Bjerkandera adusta* (Choi et al. 2014) and *Porostereum spadiceum* (Tigini et al. 2013). Interestingly, our study shows the ability of some isolates belonging to ascomycete group to decolorize textile dyes. Only, few studies reported the capacity of ascomycetes in textiles dyes decolorization (Ashrafi et al. 2013).

### Taxonomical groups of lignin degrading fungi

All taxa were taxonomically identified using BLAST and phylogenetic analysis, selecting matches with similarity higher than 98 % to sequences in the GenBank database (*E*-value < 0.05). However, 3 taxa presented low matching similarity (<98 %). Those isolates that were identified with a similarity less than 98 % were; CLBE 1 with 82 % identity with *P. macrostoma*, A3 with 94 % similarity to *T. versicolor* and BS25 with 80 % similarity to *P. spadiceum*. CLBE 24 matched with 99 % similarity an uncultured fungus not yet described (Table 1).

The 51 fungal isolates obtained in this study were classified into different taxa, representing 25 fungal genera and 29 species. Most of the isolates were found to belong to the phylum Ascomycota (67 %), some Basidiomycota (29 %) and only few Mucoromycotina (2 %) and non-identified uncultured fungi (2 %, Table 2). Ascomycetes found in this study derived from the orders Hypocraceales (27 %), Eurotiales (14 %), Pleosporales (14 % strains), Sordariales (2 %), Capnodiales (2 %), Coniochaetales (6 %) and Botryosphaeriales (2 %). Some researchers suggested a main contribution of ascomycetes in wood degradation (Nilsson and Daniel 1989; Michelotti et al. 2012). Guu et al. (2007) reported nineteen fungal isolates of the family of *Nectriaceae* collected from forests in Taiwan.

Additionally, a wide distribution of white-rot fungi throughout basidiomycetes group was also observed (29 %). Within basidiomycetes, the order polyporales was the most represented (25 %) followed by gloeophyllales (4 %). Among wood-rotting fungi, white-rot basidiomycetes are the most efficient in extensive lignin degradation (Blanchette et al. 1992). Brown-rot fungi presented by gloeophyllales order cause also the most destructive type of decay in wooden structures, although their biodegradation mechanisms are still relatively unknown (Schilling et al. 2009).

A general phylogenetic tree was generated with sequences obtained from the amplification of the ITS rDNA region and some sequences from the GenBank database (Fig. 1). Duplicates as well as diverging sequences were deleted to make it possible to draw the tree. It was observed that Polyporales (13 isolates) followed by Pleosporales and Eurotiales (7 isolates each), were the most represented orders in the tree.

**Fig. 2:**
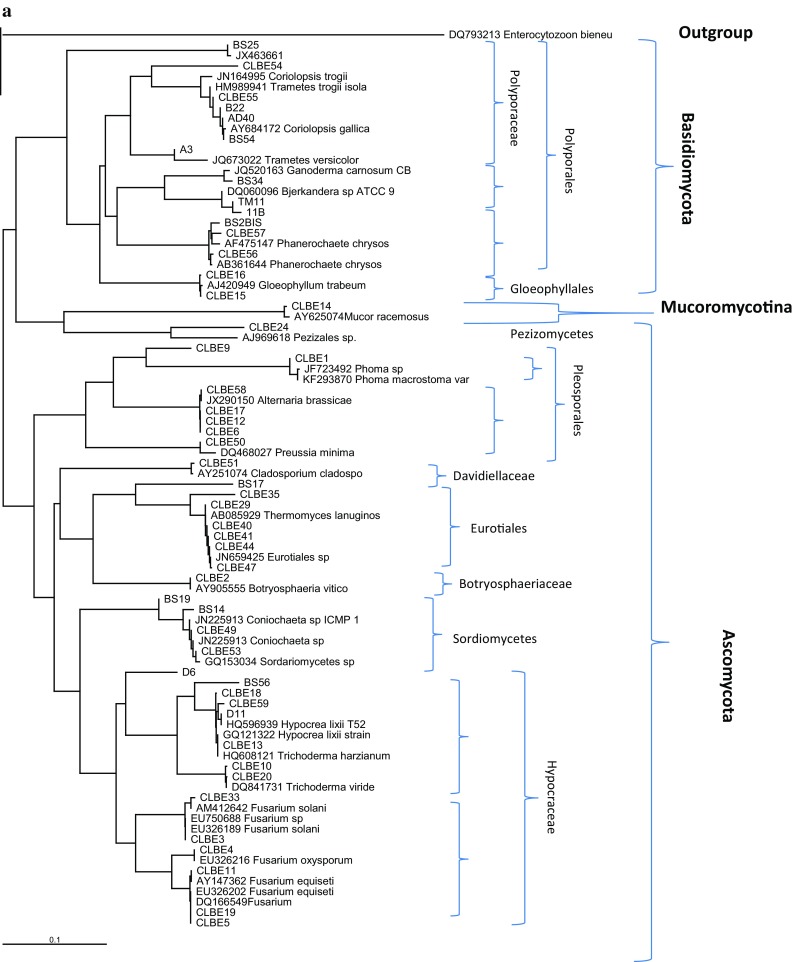

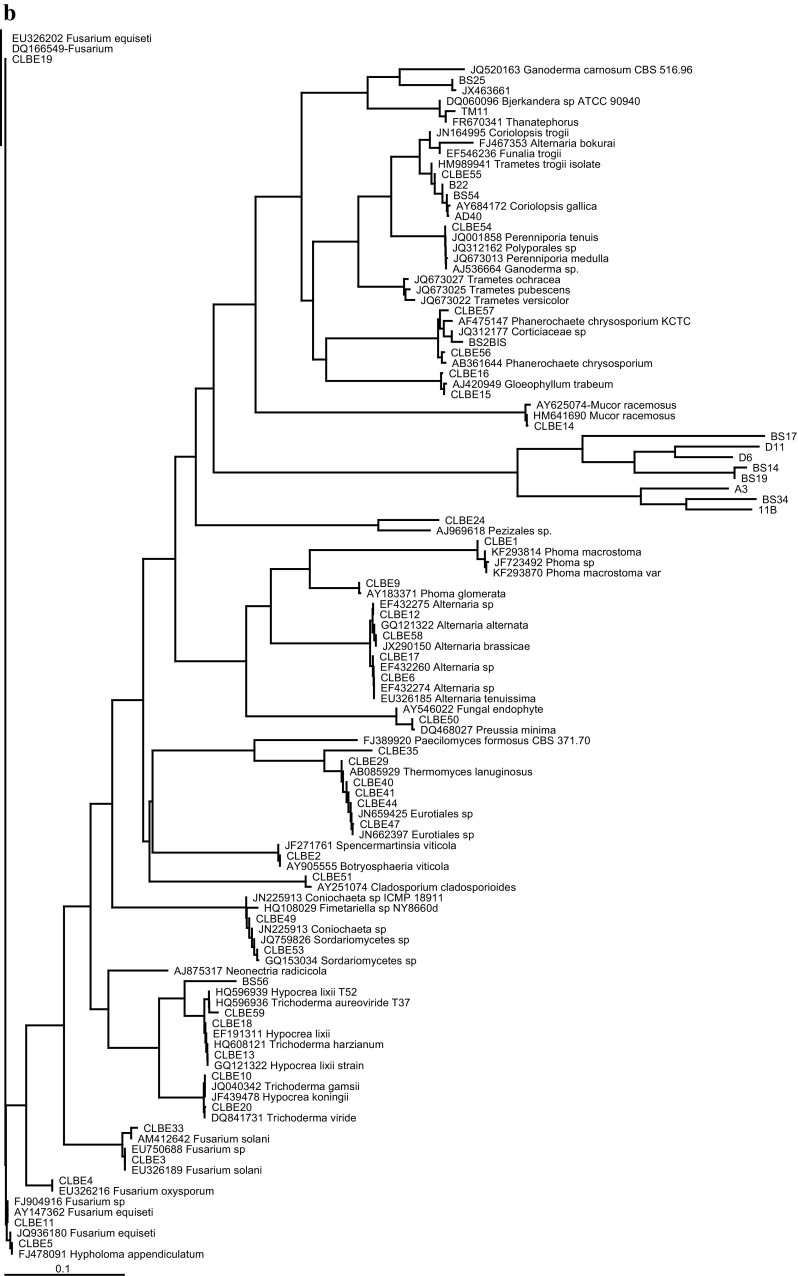
Phylogenetic relationships of recovered fungi with selected database sequences based on ITS rDNA sequences. **a** The phylogram represents a maximum likelihood tree based on analyses of 43 taxa under the HKY model. **b** The tree was rooted with Microsporidia as outgroup. Bootstrap values higher than or equal to 50 % (1000 replicates) are shown at each branches

Strain BS25 is closely related to *P. spadiceum* (JX463661.1) and *Ganoderma carnosum* (JQ520163.1) (80 % similarity). This suggests that more sampling of environmental fungi and their ITS barcoding is necessary to allow accurate identifications of strains collected in surveys similar to this study. Blast search results showed that many genera such as: *Trametes*/*Coriolopsis*, have high similarity in DNA sequences suggesting that phylogenetic analysis is necessary to ascertain the phylogenetic positions of the isolates. Our phylogenetic analysis confirmed the affiliation of most species using ClustalW for alignment and Neighbour joining (Fig. 2).

Figure 2 presents the phylogenetic affinities of 51 Tunisian isolates with some closely related taxa retrieved from GenBank. Our phylogeny confirms a close relationship of BS54, AD 40, CLBE55 and BS22 species to the members of the genus *Coriolopsis*. Despite a very close similarity in DNA sequences and phylogenetic affiliation to *C. trogii* (CLBE55, AD40 and BS22), BS54 are confirmed to be *Coriolopsis gallica.* The genus *Coriolopsis*, as currently defined, is polyphyletic, with the type species as part of the Trametoid clade and at least two additional lineages occurring in the core polyporoid clade.

## Conclusions

Our findings contribute to the discovery of new fungal strains involved in lignocellulose degradation. The isolated strains are diverse and show a wide spectrum of species demonstrating the wide biodiversity of Tunisian forests. The newly isolated fungal strains belong to different taxonomic groups with various enzymatic activities: amylases, proteases, lipases and laccases, making their further study very interesting for industrial and environmental applications. Our results show that this new collection of isolates had a certain lignin degradation capacity and provide a new resource of microorganisms for dye decolorization.

## Electronic supplementary material

Below is the link to the electronic supplementary material.

Supplementary material 1 (DOCX 18 kb)

Supplementary material 2 (TIFF 104 kb)

Supplementary material 3 (TIFF 150 kb)

Supplementary material 4 (TIFF 126 kb)

## References

[CR1] Ashrafi SD, Rezaei S, Forootanfar H, Mahvi AH, Faramarzi MA (2013). The enzymatic decolorization and detoxification of synthetic dyes by the laccase from a soil-isolated ascomycete, *Paraconiothyrium variabile*. Int Biodeterior Biodegrad.

[CR2] Barriuso J, Prieto A, Martínez MJ (2013). Fungal genomes mining to discover novel sterol esterases and lipases as catalysts. BMC Genom.

[CR3] Blanchette RA, Burnes TA, Eerdmans MM, Akhtar M (1992). Evaluating isolates of *Phanerochaete chrysosporium* and *Ceriporiopsis subvermispora* for use in biological pulping processes. Holzforschung.

[CR4] Caporale C, Garzillo AM, Caruso C, Buinocore V (1996). Characterization of extracellular proteases from *Trametes troggi*. Phytochemistry.

[CR5] Cheong S, Yeo S, Song H-G, Choi HT (2006). Determination of laccase gene expression during degradation of 2,4,6-trinitrotoluene and its catabolic intermediates in *Trametes versicolor*. Microbiol Res.

[CR6] Cho NS, Wilkolazka AJ, Staszczak M, Cho HY, Ohga S (2009). The role of laccase from white rot fungi to stress conditions. J Fac Agr Kyushu Univ.

[CR7] Choi YS, Kim JJ, Kim MJ, Imamura Y, Yoshimura T, Kim GH (2012). Fungal biodegradation of CCA-treated wood and removal of its metal components. Chemosphere.

[CR8] Choi YS, Seo JY, Lee H, Yoo J, Jung J, Kim JJ, Kim GH (2014). Decolorization and detoxification of wastewater containing industrial dyes by *Bjerkandera adusta* KUC9065. Water Air soil Pollut.

[CR9] Colao M, Garzillo Ch, Buonocore AM, Schiesser VA, Ruzzi M (2003). Primary structure and transcription analysis of a laccase-encoding gene from the basidiomycete *Trametes trogii*. Appl Microbiol Biotechnol.

[CR10] Colussi F, Serpa V, Delabona PDS, Manzine LR, Voltatodio ML, Alves R, Mello BL, Pereira N, Farinas CS, Golubev AM, Santos MA, Polikarpov I (2011). Purification, and biochemical and biophysical characterization of cellobiohydrolase I from *Trichoderma harzianum* IOC 3844. J Microbiol Biotechnol.

[CR11] Cruz-Ramírez MG, Rivera-Ríos JM, Téllez-Jurado A, Maqueda Gálvez AP, Mercado-Flores Y, Arana-Cuenca A (2012). Screening for thermotolerant ligninolytic fungi with laccase, lipase, and protease activity isolated in Mexico. J Environ Manag.

[CR12] Daâssi D, Frikha F, Zouari-Mechichi H, Belbahri L, Woodward S, Mechichi T (2012). Application of response surface methodology to optimize decolourization of dyes by the laccase-mediator system. J Environ Manag.

[CR13] Daâssi D, Mechichi T, Nasri M, Rodríguez-Couto S (2013). Decolorization of the metal textile dye Lanaset Grey G by immobilized white-rot fungi. J Environ Manag.

[CR14] Dashtban M, Schraft H, Syed TA, Qin W (2010). Fungal biodegradation and enzymatic modification of lignin. Int J Biochem Mol Biol.

[CR15] Dass SB, Dosoretz CG, Reddy CA, Grethlein HE (1995). Extracellular proteases produced by the wood-degrading *fungus Phanerochaete chrysosporium* under ligninolytic and non-ligninolytic conditions. Arch Microbiol.

[CR16] de Castro AM, Ferreira MC, da Cruz JC, Pedro KC, Carvalho DF, Leite SG, Pereira N (2010). High-yield endoglucanase production by *Trichoderma Harzianum* IOC-3844 cultivated in pretreated sugarcane mill byproduct. Enzyme Res.

[CR17] Diwaniyan S, Kharb D, Raghukumar C, Kuhad RC (2010). Decolorization of synthetic dyes and textile effluents by basidiomycetous fungi. Water Air soil Pollut.

[CR18] Eriksson K-EL, Blanchette RA, Ander P (1990). Microbial and enzymatic degradation of wood components.

[CR19] Fackler K, Gradinger C, Hinterstoisser B, Messner K, Schwanninger M (2006). Lignin degradation by white rot fungi on spruce wood shavings during short-time solid-state fermentations monitored by near infrared spectroscopy. Enzyme Microbial Technol.

[CR20] Gnanasalomi VDV, Gnanadoss JJ (2013). Molecular characterization and phylogenetic analysis of laccase producing fungal isolates with dye decolourizing potential. Res Biotechnol.

[CR21] Gomaa OM (2012). Ethanol induced response in *Phanerochaete chrysosporium* and its role in the decolorization of triarylmethane dye. Ann Microbiol.

[CR22] Guu JR, Ju YM, Hsieh HJ (2007). Nectriaceous fungi collected from forests in Taiwan. Bot Stud.

[CR23] Hatakka A (1994). Lignin-modifying enzymes from selected White-rot fungi—production and role in lignin degradation. FEMS Microbiol Rev.

[CR24] Hatakka A, Hammel KE, Hofrichter M (2010). Fungal biodegradation of lignocelluloses. The mycota X industrial applications.

[CR25] Hawksworth DL (2001). The magnitude of fungal diversity: the 1.5 million species estimate revisited. Mycol Res.

[CR26] Hussain A, Shrivastav A, Jain SK, Baghel RK, Rani S, Agrawale MK (2012). Cellulolytic enzymatic activity of soft rot filamentous fungi *Paecilomyces variotii*. Adv Biores.

[CR27] Jallouli R, Khrouf F, Fendri A, Mechichi T, Gargouri Y, Bezzine S (2012). Purification and biochemical characterization of a novel alkaline (Phospho)lipase from a newly isolated *Fusarium solani* strain. Appl Biochem Biotechnol.

[CR28] Khlifi R, Sayadi S, Belbahri L, Woodward S, Mechichi T (2009). Effect of HBT on the stability of laccase during the decolourization of textile wastewaters. J Chem Technol Biotechnol.

[CR29] Kiiskinen LL, Viikari L, Kruus K (2002). Purification and characterisation of a novel laccase from the ascomycete *Melanocarpus albomyces*. Appl Microbiol Biotechnol.

[CR30] Kiiskinen LL, Ratto M, Kruus K (2004). Screening for novel laccase-producing microbes. J Appl Microbiol.

[CR31] Kouker G, Jaeger KE (1987). Specific and sensitive plate assay for bacterial lipases. Appl Environ Microbiol.

[CR32] Kovalenko GA, Beklemishev AB, Perminova LV, Chuenko TV, Ivanov ID, Moiseenkov SI, Kuznetsov VL (2013). Recombinant strain producing thermostable lipase from *Thermomyces lanuginosus* immobilized into nanocarbon silica matrices and properties of the prepared biocatalyzers. Prikl Biokhim Mikrobiol.

[CR33] Lee H, Jang Y, Kim JM, Kim GH, Kim JJ (2013). White-rot fungus *Merulius tremellosus* KUC9161 identified as an effective degrader of polycyclic aromatic hydrocarbons. J Basic Microbiol.

[CR34] Leonowicz A, Matuszewska A, Luterek J, Ziegenhagen D, Wojtas-Wasilewska M, Cho NS, Hofrichter M (1999). Biodegradation of lignin by white-rot fungi. Fungal Genet Biol.

[CR73] Li S, Gao H, Zhang J, Li Y, Peng B, Zhou Z (2011). Determination of insecticides in water using in situ
halide exchange reaction-assisted ionic liquid dispersive liquid–liquid microextraction followed by highperformance liquid chromatography. J Sep Sci.

[CR35] Ljungdahl LG (2008). The cellulase/hemicellulase system of the anaerobic fungus Orpinomyces PC-2 and aspects of its applied use. Ann N Y Acad Sci.

[CR36] Ma L, Zhuo R, Liu H, Yu D, Jiang M, Zhang X, Yang Y (2014). Efficient decolorization and detoxification of the sulfonated azo dye Reactive Orange 16 and simulated textile wastewater containing Reactive Orange 16 by the white-rot fungus *Ganoderma* sp. En3 isolated from the forest of Tzu-chin Mountain in China. Biochem Eng J.

[CR37] Machado KMG, Matheus DR, Bononi VLR (2005). Ligninolytic enzymes production and Remazol Brilliant Blue R decolorization by tropical Brazilian basidiomycetes fungi. Braz J Microbiol.

[CR38] Martínez AT, Speranza M, Ruiz-Dueñas J, Ferreira P, Camarero S, Guillén F, Martínez MJ, Gutiérrez A, del Río JC (2005). Biodegradation of lignocellulosics: microbial, chemical, and enzymatic aspects of the fungal attack of lignin. Int Microbiol.

[CR39] Martínez AT, Ruiz-Dueñas FJ, Martínez MJ, del Río JC, Gutiérrez A (2009). Enzymatic delignification of plant cell wall: from nature to mill. Curr Opin Biotechnol.

[CR40] Mayerhofer HJ, Marshall RT, White CH, Lu M (1973). Characterization of a heat stable protease of *Pseudomonas fluorescens P23*. Appl Microbiol.

[CR41] Michelin M, Silva TM, Benassi VM, Peixoto-Nogueira SC, Moraes LA, Leão JM, Jorge JA, Terenzi HF, Polizeli Mde L (2010). Purification and characterization of a thermostable α-amylase produced by the fungus *Paecilomyces variotii*. Carbohydr Res.

[CR42] Michelotti S, Guglielmo F, Gonthier P (2012). Detection of the wood decay ascomycete *Kretzschmaria deusta* in urban maple trees in Italy. J Plant Pathol.

[CR43] Moldes D, Cadena EM, Vidal T (2010). Biobleaching of eucalypt kraft pulp with a two laccase-mediator stages sequence. Bioresour Technol.

[CR44] Nilsson T, Daniel G (1989). Chemistry and microscopy of wood decay by some higher ascomycetes. Holzforschung.

[CR45] Obruca S, Marova I, Matouskova P, Haronikova A, Lichnova A (2012). Production of lignocellulose-degrading enzymes employing *Fusarium solani* F-552. Folia Microbiol.

[CR46] Orth AB, Tien M, Esser K, Lemke PA (1995). Biotechnology of lignin degradation. The mycota. II. Genetics and biotechnology.

[CR47] Peláez F, Martínez MJ, Martínez AT (1995). Screening of 68 species of basidiomycetes for enzymes involved in lignin degradation. Mycol Res.

[CR48] Reddy CA (1995). The potential for white-rot fungi in the treatment of pollutants. Curr Opin Biotechnol.

[CR49] Rossello Mora R, Amman R (2001). The species concept for prokaryotes. Microbiol Res.

[CR50] Ruiz-Dueñas FJ, Martínez AT (2009). Microbial degradation of lignin: how a bulky recalcitrant polymer is efficiently recycled in nature and how we can take advantage of this. Microbial Biotechnol.

[CR51] Ryu WY, Jang MY, Cho MH (2003). The selective visualization of lignin peroxidase, manganese peroxidase and laccase, produced by white rot fungi on solid media. Biotechnol Bioprocess Eng.

[CR52] Saitou N, Nei M (1987). The neighbor-joining method: a new method for reconstructing phylogenetic trees. Mol Biol Evol.

[CR53] Sakthivel M, Karthikeyan N, Jayaveny R, Palani P (2010). Optimization of culture conditions for the production of extracellular cellulase from *Corynebacterium lipophiloflavum*. J Ecobiotechnol.

[CR54] Sato A, Watanabe T, Watanabe Y, Harazono K, Fukatsu T (2002). Screening for basidiomycetous fungi capable of degrading 2,7-dichlorodibenzo-p-dioxin. FEMS Microbiol Lett.

[CR55] Schilling JS, Tewalt JP, Duncan SM (2009). Synergy between pretreatment lignocellulose modifications and saccharification efficiency in two brown rot fungal systems. Appl Microbiol Biotechnol.

[CR56] Schoemarker HE (1990). On the chemistry of lignin degradation. Recl Trav Chim Pays Bas.

[CR57] Schoemarker HE, Tuor U, Muheim A, Schmidt HWH, Leisola MSA, Betts WB (1991). White-rot degradation of lignin and xenobiotics. Biodegradation natural and synthetic materials.

[CR58] Selvam K, Shanmuga Priya M, Sivaraj C, Arungandhi K (2012). Identification and Screening of wood rot fungi from Western Ghats area of South India. Int J Chem Tech Res.

[CR59] Shimada M, Higuchi T, Hon DNS, Shiraishi N (1991). Microbial, enzymatic and biomimetic degradation of lignin. Wood and cellulosic chemistry.

[CR60] Sigoillot JC, Berrin JG, Bey M, Lesage-Meessen L, Levasseur A, Lomascolo A, Record E, Uzan-Boukhris E (2012). Fungal strategies for lignin degradation. Adv Bot Res.

[CR61] Silva CMMS, Melo IS, Oliveira PR (2005). Ligninolytic enzyme production by *Ganoderma *spp. Enzyme Microb Technol.

[CR62] Singh NGKP, Modi DR (2013). Screening of soil fungi for α-amylase activity. J Recent Adv Appl Sci.

[CR63] Staszczak M, Nowak G (1984). Proteinase pattern in *Trametes versicolor* in response to carbon and nitrogen starvation. Biochem J.

[CR64] Sugano Y (2009). DyP-type peroxidases comprise a novel heme peroxidase family. Cell Mol Life Sci.

[CR65] Taylor JW (1995). Making the Deuteromycota redundant: a practical integration of mitosporic and meiosporic fungi. Can J.

[CR66] Thompson JD, Higgins DG, Gibson TJ (1994). CLUSTAL W: improving the sensitivity of progressive multiple sequence alignment through sequence weighting, position-specific gap penalties and weight matrix choice. Nucleic Acids Res.

[CR67] Thurston CF (1994). The structure and function of fungal laccase. Microbiology.

[CR68] Tigini V, Spina F, Romagnolo A, Progione V, Varese GC (2013). Effective biological treatment of landfill leachates by means of selected white rot fungi. Chem Eng Trans.

[CR69] Xiao P, Mori T, Kondo R (2011). Biotransformation of the organochlorine pesticide trans-chlordane by wood-rot fungi. New Biotechnol.

[CR70] Xiong X, Wen C, Bai Y, Oian Y (2008). Effects of culture conditions on ligninolytic enzymes and proteases production by *Phanerochaete chrysosporium* in air. J Environ Sci China.

[CR71] Yoon JJ, Cha CJ, Kim YS, Son DW, Kim YK (2007). The brown-rot basidiomycete *Fomitopsis palustris* has the endo-glucanases capable of degrading microcrystalline cellulose. J Microbiol Biotechnol.

[CR72] Zouari-Mechichi H, Mechichi T, Dhouib A, Sayadi S, Martınez AT, Martınez MJ (2006). Laccase purification and characterization from *Trametes trogii* isolated in Tunisia: decolorization of textile dyes by the purified enzyme. Enzyme Microb Technol.

