# Supplementary material for: Phylogenetic and metabolic diversity of Tunisian forest wood-degrading fungi: a wealth of novelties and opportunities for biotechnology
Source: 3 Biotech. 2016 Feb 4;6(1):46. doi: 10.1007/s13205-015-0356-8 (PMC4742418; doi:10.1007/s13205-015-0356-8)
Supplement: Supplementary file 1 — Supplementary material 1 (DOCX 18 kb) [file 13205_2015_356_MOESM1_ESM.docx]

SUPPLEMENTARY FIGURES CAPTIONS

Absorbance spectrum of: (a) RBBR (b) RB-5 and (c) GL by laccase from *Coriolopsis gallica (BS54)*
