# Supplementary figures and images for: Phylogenetic and metabolic diversity of Tunisian forest wood-degrading fungi: a wealth of novelties and opportunities for biotechnology
Source: 3 Biotech. 2016 Feb 4;6(1):46. doi: 10.1007/s13205-015-0356-8 (PMC4742418; doi:10.1007/s13205-015-0356-8)

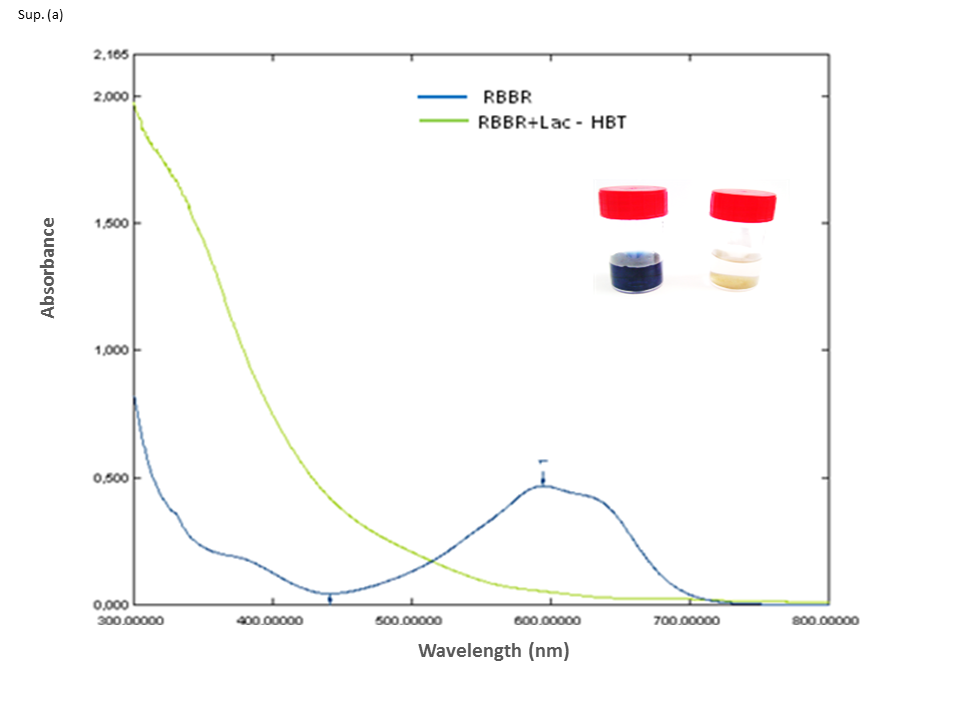

Supplement: Supplementary file 2 — Supplementary material 2 (TIFF 104 kb) [file 13205_2015_356_MOESM2_ESM.tif]

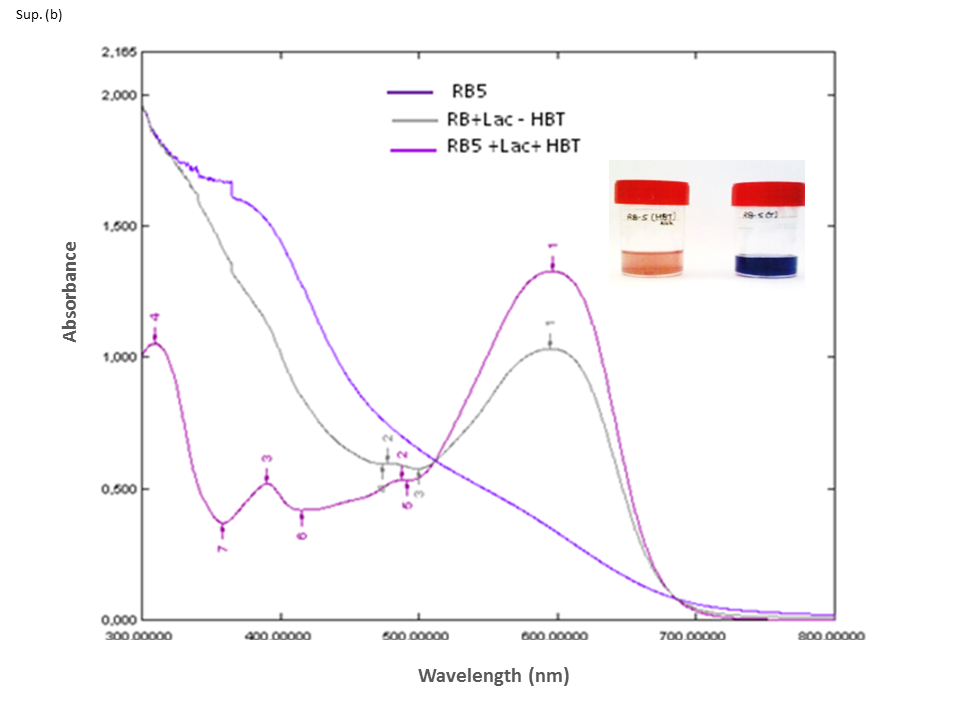

Supplement: Supplementary file 3 — Supplementary material 3 (TIFF 150 kb) [file 13205_2015_356_MOESM3_ESM.tif]

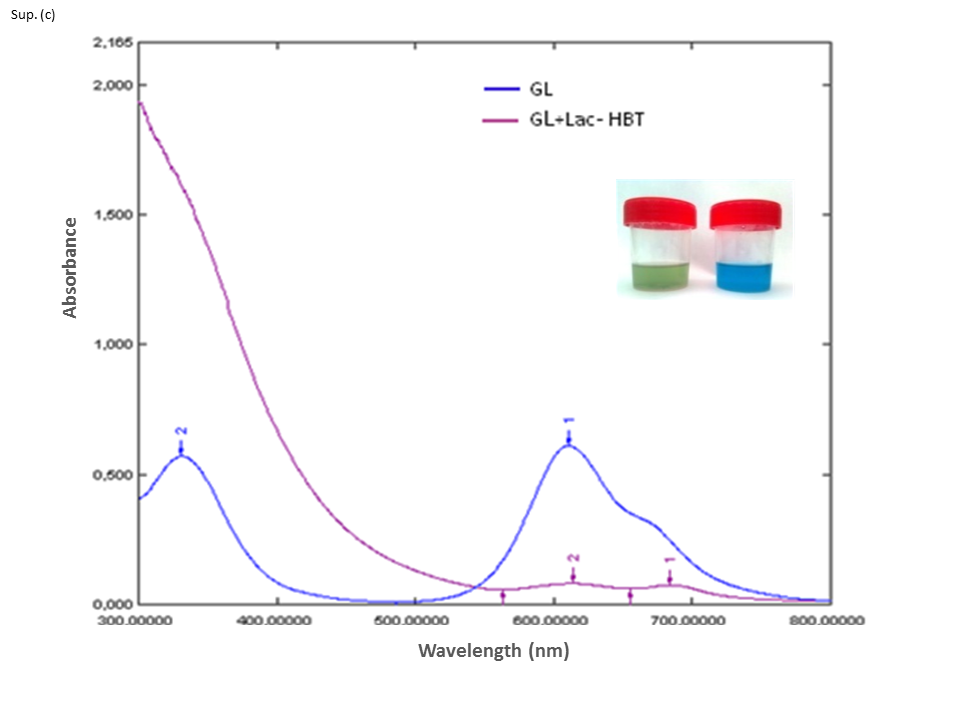

Supplement: Supplementary file 4 — Supplementary material 4 (TIFF 126 kb) [file 13205_2015_356_MOESM4_ESM.tif]
